# Long non-coding RNAs in osteosarcoma

**DOI:** 10.18632/oncotarget.14726

**Published:** 2017-01-18

**Authors:** Ruiling Chen, Gangyang Wang, Ying Zheng, Yingqi Hua, Zhengdong Cai

**Affiliations:** ^1^ Department of Orthopaedics, Shanghai Bone Tumor Institute, Shanghai General Hospital, Shanghai Jiao Tong University School of Medicine, Shanghai, China

**Keywords:** osteosarcoma, lncRNA, pathogenesis, biomarkers, therapeutic targets

## Abstract

Long non-coding RNAs (lncRNAs) with more than 200 nuleotides, have been explored to participate in various cancer types including osteosarcoma (OS), which is the most common kind of primary bone tumors with high morbidity in infants and adolescents. These oncogenic or tumor suppressive lncRNAs regulate OS pathogenesis, such as cell growth, proliferation, invasion, migration, metastasis and cell apoptosis, serve as independent prognostic biomarkers or play a significant role in multidrug resistance (MDR) in OS cells. In this review, we attempt to dissect the participation of lncRNAs in pathogenesis of OS and their potential clinical values, and also provide an outlook for viable biomarkers and therapeutic targets in OS.

## INTRODUCTION

Osteosarcoma(OS) is the most common primary malignant bone tumor in childhood and adolescence, the incidence of which is about (1-3)/1000, 000 per year throughout the world, with highly aggressive and early systemic metastasis [1–4]. Approximately 10%-25% of patients occurred lung metastasis, meanwhile pulmonary damage is the most prominent reason for OS-caused death [5]. With the application of chemotherapeutics, the combination of surgery resection and multi-chemotherapy has clinically become a standard treatment strategy for almost all OS patients and significantly improved patients’ survival [6]. The cure rates of OS patients with focal tumor are elevated from less than 20% before 1970s to present levels of 65-75% [7–9], while only 11%-30% of patients with matastasitic OS can survive [10–13]. Besides, many OS patients present resistance to available chemotherapeutics and then die of widespread metastasis and tumor relapse, which is a significant obstacle for successful OS treatments [14]. Despite of great efforts to search for novel therapeutic approaches, the overall survival of patients with OS has reached a plateau in recent 30 years [15]. Till now, we have well understood the biological characteristic of OS, but there still exist a large scale of fuzzy regions in exploration of molecular mechanisms involved in OS origination, metastasis, and chemoresistance. Therefore, candidate molecules as diagnostic or prognostic biomarkers and therapeutic targets are expected to be identified and may profoundly improve therapeutic efficacy and clinical outcomes for patients with OS. Inspiringly, some long non-coding RNAs (lncRNAs) have recently been reported to exert a key role in OS.

LncRNAs are transcripts that are numerous and unable to translate proteins in the intracellular space. It has been revealed that more than 90% of the human genome DNA is thought to be transcribed, while only about 2% of it can encode proteins. Majority of these transcribed RNAs are termed as non-coding RNAs (ncRNAs) [16, 17]. According to their transcript length, ncRNAs can be classified into 2 groups: small non-coding RNAs (small ncRNAs) composed of less than 200 nucleotides and long non-coding RNAs (lncRNAs) consisting of more than 200 nucleotides. The small ncRNAs [18] which include microRNAs (miRNAs), small interfering RNAs (siRNAs), piwi-interacting RNAs, transfer RNAs, and some ribosomal RNA, have been reported to function in the tumorigenesis, metastasis and chemoresistance of OS [19, 20]. However, lncRNAs have gained much less attention. In that lncRNAs account for over 70% of ncRNAs [21], we may predict that they contain much more genetic information which is needed to be explored. Thus, more and more researchers set to seek for associated lncRNAs in the pathogenesis or drug resistance mechanisms of OS, in order to discover the unknown functions of lncRNAs.

During recent few years, few but noteworthy studies concerning functions of lncRNAs in OS pathogenesis and drug resistance have successively been reported, which might provide valuable biomarkers for diagnosis or prognosis and show great promise for exploring novel therapeutic approaches based on lncRNAs. Therefore, great benefits will be brought to improve the quality of life and overall survival for patients with OS. The present review will dissect the involvement of lncRNAs in pathogenesis of OS and their potential clinical application, and show possible anticipation of viable biomarkers and therapeutic targets in OS.

## WHAT ARE THE LNCRNAS

**Figure 1:**
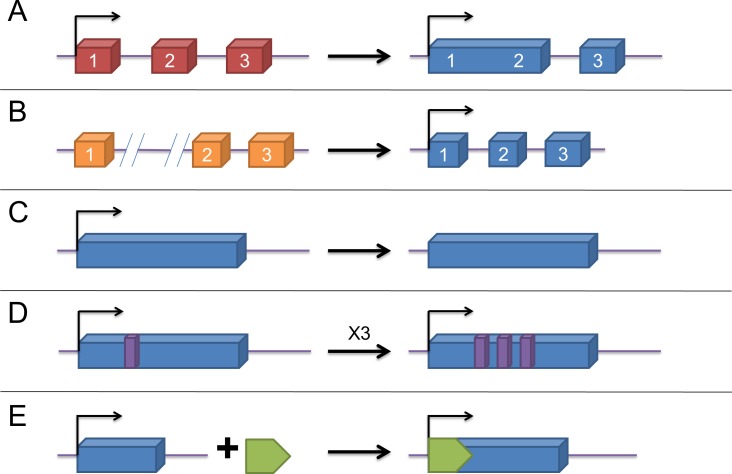
The origins of lncRNAs **A**. a lncRNA is transformed from a protein-coding gene with structural damage; **B**. two abreast non-transcribed regions generate a lncRNA after chromosomal rearrangement; **C**. copy of a noncoding gene by retrotransposition forms a lncRNA without protein-coding ability ; **D**. a lncRNA with adjacent repeats derives from tandem duplication events; **E**. a functional lncRNA with insertion of a transposon.

With over 200 nucleotides in length, lncRNAs are characteristic of lack of protein-coding ability and were originally discovered in the transcripts of mice. [22] Later on, they were found to have five different origins: (A) a protein-coding gene gains structural damage and is transformed into a lncRNA; (B) by chromosomal rearrangement, two non-transcribed regions are in juxtaposition and generate a lncRNA with multiple exons; (C)duplication of a noncoding gene through retrotransposition forms either a functional retrogene or a nonfunctional retropseudogene, both without encoding proteins; (D)two tandem duplication events give rise to the adjacent repeats within a noncoding RNA; and (E)insertion of a transposon composes a functional lncRNA (Figure 1) [23]. Based on their location in regard to protein-coding genes, they can be divided as follows: intergenic lncRNAs (lincRNAs), intronic lncRNAs (antisense/sense), exonic lncRNAs (antisense/sense), and overlapping lncRNAs (antisense/sense) [24, 25]. Initially, they were considered as transcriptional “noise” due to numerous transcripts [26]. However, with in-depth studies in recent years, they have been found to possess plenty of biological functions, including gene expression regulation [23, 27] at the epigenetic, transcriptional and posttranscriptional levels, and involvement in various cellular processes, which contain proliferation, differentiation, cell cycle progression, growth and apoptosis [28, 29]. Recently, considerable researches have shown that lncRNAs, serving as pivotal regulators and acting as oncogenes or tumor suppressors, play a crucial role in many different types of cancers, with the capacity to participate in tumor development, metastasis and chemoresistance. [30–34]

**Figure 2:**
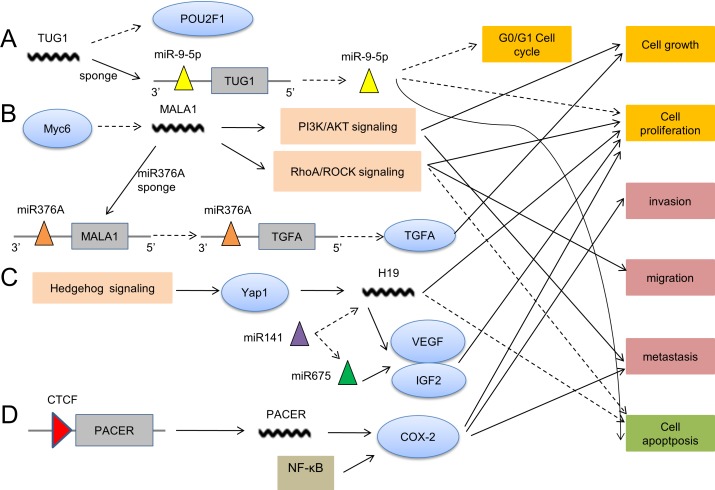
The action mechanisms of lncRNAs in OS pathogenesis **A**. TUG1 decreases *POU2F1* expression and sponges miR-9-5p to promote cell proliferation and cell cycle, but inhibits cell apoptosis. **B**. MALAT1 plays a role in OS pathogenesis *via* the involvement of PI3K/AKT signaling, RhoA/ROCK signaling and sponging miR376A. **C**. H19 is upregulated by Hedgehog signaling to facilitate cell proliferation and suppress apoptosis. H19 can also interact with miRNA. **D**. With the binding of CTCF (CCCTC-binding factor), PACER functions by targeting COX-2 which is also regulated by NF-κB.

**Figure 3:**
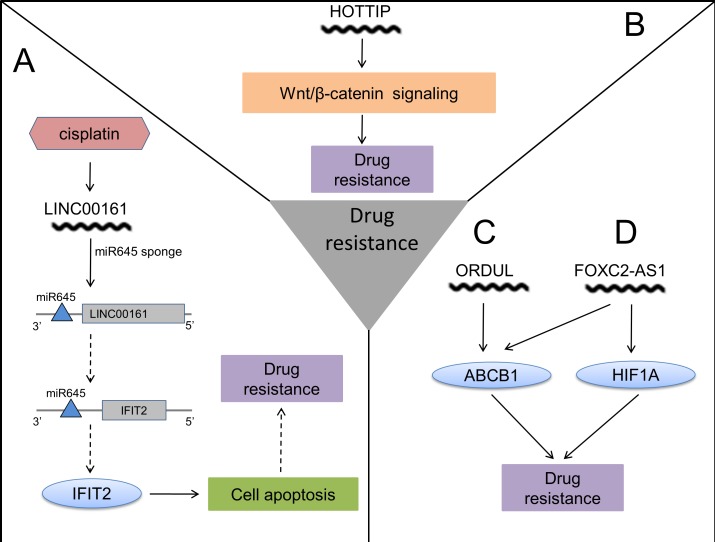
The action mechanisms of lncRNAs in OS chemoresistance **A**. Cisplatin induces expression of LINC00161, which sponges miR645 to reverse drug resistance. **B**. HOTTIP contributes to drug resistance through activating Wnt/β-catenin signaling. **C**. ORDUL increases drug resistance by elevating ABCB1. **D**. High expression of FOXC2-AS1 causes drug resistance with increased ABCB1 and HIF1A. (The solid arrow represents promoting and the dashed arrow represents inhibiting.)

## ROLES OF LNCRNAS IN OS PATHOGENESIS

Ever-growing evidence has unraveled the roles of lncRNAs in the pathogenesis of multiple human diseases including osteoarthritis [35] and OS [36]. Jinping Li et al. are the first to explore the expression profile of lncRNAs in OS. In 9 human primary OSs and their paired adjacent noncancerous samples, they found a total number of 25,733 lncRNAs through microarray analysis, with 403 upregulated lncRNAs and 798 down-regulated lncRNAs. They further performed pathway analysis which indicated 34 pathways related to upregulated transcripts and 32 pathways related to downregulated transcripts [36]. Therefore, this study commendably hints the possible roles of lncRNAs as candidate biomarkers for diagnosis or prognosis and potential therapeutic targets in OS. However, the research progress of lncRNAs in OS is still preliminary, even though lncRNAs have become a hotspot of cancer researches in recent years and appealing progress has been made in lung cancer, breast cancer and gastric cancer, etc. [37–40] Excitingly, a small amout of oncogenic lncRNAs and tumor suppressive lncRNAs have been elucidated to be involved in the OS pathogenesis, including cell growth, proliferation, invasion, migration, metastasis and cell apoptosis. Herein, we summarize the known roles and functional mechanisms of the limited lncRNAs related to OS pathogenesis, which are shown in Table 1. These lncRNAs are found to function by several mechanisms: I) targeting some associated host genes like MFI2, SNHG12, PACER and BCAR4; II) involvement of signaling pathways like MALAT1, H19 and HOTTIP; III) competing endogenous RNA like TUG1, MALAT1 and H19, and so on. Intrestingly, TUG1, MEG3 and loc285194 were reported to be induced by p53 or regulate p53 expression in other diseases not in OS. However, it has been well illustrated that aberrations in p53 are common in OS [41], thus lncRNAs associated with p53 may be important regulators. Since both miRNA and lncRNAs can serve as oncogenes or tumor suppressors in OS [42], the interaction between lncRNAs and miRNAs implies the existence of competitive RNA regulatory network, which will further complement underlying molecular components in the pathogenesis of OS.

**Table 1:** Roles and function mechanisms of lncRNAs in osteosarcoma

LncRNA	Expression	Role of lncRNA	Function mechanism	Reference
TUG1	↑	promotes cell proliferation, inhibits apoptosis, inhibits G0/G1 cell cycle arrest	sponges miR-9-5p:downregulates *POU2F1* expression	[29, 49–51, 87]
		associated with tumor size, clinical stage and post-operative chemotherapy	downregulates *POU2F1* expression	
		correlated with poor prognosis and disease status		
		an independent prognostic indicator for overall survival		
HOTAIR	↑	promotes cell proliferation, invasion and secrection of MMP2 and MMP9	—	[62, 67, 68]
MALAT1	↑	promotes cell proliferation and meatastasis	activates PI3K/AKT signaling pathway;	[53–56]
			involvement of RhoA/ROCK Pathway	
			MALAT1/MIR376A/TGFA axis	
			regulated by Myc-6	
H19	↑	promotes cell proliferation, inhibites apoptosis	interacts with miR-141	[60, 61]
		induces osteosarcoma development	involvement of Hedgehog signaling	
HULC	↑	correlated with clinical stage, and distant metastasis, but not in another study	—	[78, 79]
		correlated with shorter overall survival		
SNHG12	↑	promotes cell proliferation and migration	upregulates angiomotin gene expression	[45]
MFI2	↑	promotes cell proliferation, suppresses apoptosis	upregulates *FOXP4* expression	[46]
PACER	↑	promotes cell proliferation and metastasis	activates *COX-2* gene in a NFκB-dependent way	[47]
BCAR4	↑	correlated with large tumor size, advanced Enneking stage, lung metastasis,	activates *GLI2*-dependent gene transcription	[80]
		and poor prognosis		
HNF1A-AS1	↑	associated with clinical stage, distant metastasis, and poor overall survival	activates Wnt/β-catenin signaling pathway	[44]
BANCR	↑	associated with large tumor size, distant metastasis, and advanced clinical stage	—	[27, 86, 110]
		an independent predictor of poor survival		
UCA1	↑	correlated with large tumor size, high tumor grade, distant metastasis,	—	[81]
		and advanced clinical stage		
		an independent unfavorable prognostic factor		
91H	↑	correlated with advanced clinical stage, chemotherapy after surgery,	—	[88]
		and tumor size >5 cm		
		an independent prognostic factor for overall survival		
ODRUL	↑	contributes to doxorubicin resistance	induces multidrug resistance-related *ABCB1*expression	[77]
HOTTIP	↑	correlated with advanced clinical stage, distant metastasis and poor prognosis	interacts with WDR5/MLL complex	[83, 108, 109]
		increases cisplatin resistance	activates Wnt/β-catenin signaling pathway	
FOXC2-AS1	↑	influences tumor angiogenesis and metastasis	upregulates *ABCB1*, *HIF1A* and *FOXC2* expression	[20]
		associated with poor chemoresponse and longer survival time		
		contributes to doxorubicin resistance		
LINC00161	↑	increases cisplatin-induced apoptosis	sponges endogenous miR-645	[107]
		reverses cisplatin resistance	upregulates *IFIT2*	
ZEB1-AS1	↑	promotes cell proliferation and migration	binds and recruits p300 to the *ZEB1* promoter region,	[48]
		correlated with large tumor size, progressed Enneking stage, tumor metastasis,	activates *ZEB1* transcription	
		worse recurrence-free and overall survival		
loc285194	↓	promotes cell growth	induced by *p53*,interact with miR-211	[70, 71]
MEG3	↓	associated with poor overall survival	positively regulates *p53* expression	[84, 85]
TUSC7	↓	promotes cell proliferation and increases colony formation in vitro	—	[43]
HIF2PUT	↓	promotes cell proliferation and migration	positively correlated with *HIF-2α* expression	[94]
		decreases the percentage of CD133 expressing cells		
		impaires the osteosarcoma stem sphere-forming ability of the MG63 cells		

## LNCRNAS IN CELL GROWTH AND PROLIFERATION

Recent studies have reported that aberrant expression of lncRNAs led to OS cell growth and proliferation, and then facilitated OS tumorigenesis. These findings show great promise for these lncRNAs to be potential therapeutic targets for OS treatments. For instance, inhibition of tumor suppressor lncRNA TUSC7 (tumor suppressor candidate 7, TUSC7) could promote OS cell proliferation and enhance colony formation *in vitro* [43]. Down expression of HNF1A-AS1 (HNF1A-antisense 1, HNF1A-AS1), inhibited cell proliferation *via* inactivating Wnt/β-catenin pathway in OS cells [44]. Besides, some lncRNAs are found to induce OS by a mechanism of targeting associated host genes. SNHG12 (small nucleolar RNA host gene 12, SNHG12), an oncogenic lncRNA, functions in cell proliferation by upregulating expression of angiomotin gene in human OS cells [45]. MFI2 (modified frailty index 2, MFI2) plays a positive role in occurrence and development of OS by enhancing FOXP4 (forkhead box P4, FOXP4) expression [46]. PACER (P50-associated COX-2 extragenic RNA, PACER), which is regulated by DNA methylation, promotes cell proliferation in OS *via* NFκB-dependent activation of COX-2 gene [47]. Oncogenic lncRNA ZEB1-AS1 (ZEB1 Antisense 1, ZEB1-AS1) contributes to OS cells proliferation by activating ZEB1 transcription [48].

TUG1 (Taurine Up-regulated Gene 1, TUG1), a 6.7kb lncRNA located at 22q12.2, was previously suggested to be induced by p53, couple with PRC2 (polycomb repressive complex 2), and play a role in suppressing specific genes to regulate cell cycle [49]. Later on, significantly higher TUG1 levels were detected by Qiang Zhang et al. in 19 cases of OS tissues compared with matched adjacent non-tumorous tissues, and inhibition of TUG1 expression distinctly impaired proliferation of OS cells [29, 50]. Importantly, the underlying molecular mechanism of TUG1 was demonstrated in a recent article, in which TUG1 inhibited G0/G1 cell cycle arrest and facilated OS tumorigenesis *via* sponging miR-9-5p and decreasing POU2F1 (POU class 2 homeobox 1, POU2F1) expression [51], which supports the existence of competitive RNA regulatory network.

MALAT1 (metastasis-associated lung adeno-carcinoma transcript 1, MALAT1), a 8708nt lncRNA located at 11q13.1, was first identified in non-small cell lung cancer by Ji et al. in 2003 [52]. Taniguchi M et al. pointed out that Myc-6 may serve as a tumor suppressor in human OS cells partly by specifically down-regulating MALAT1, implying the potential role of MALAT1in OS development [53]. Subsequently, Dong Y et al. reported that MALAT1 played an oncogenic role to promote cell growth in OS, probably by activating PI3K/AKT signaling pathway [54]. Additionally, RhoA/ROCK Pathway was revealed to involve in the MALAT1-mediated occurrence of OS [55]. Interestingly, Wei Luo et al. discovered the involvement of miRNA in MALAT1-mediated OS. They elucidated a MALAT1/MIR376A/TGFA (transforming growth factor alpha, TGFA) axis participating in OS progression, in which MALAT1 promoted OS development by acting as endogenous miRNA sponges to repress MIR376A and increase TGFA expression [56]. These findings indicate the significant function of interaction between lncRNAs and miRNAs.

H19, a 6295nt lncRNA, lies in an imprinted region of chromosome 11 and is adjacent to IGF2 (insulin-like growth factor 2, IGF2) gene which includes a miR-675 in exon one [57]. It has been reported that lncRNA H19 can play either oncogenic or tumor suppressive role in the progression of diverse cancers [58, 59]. Recently, H19 has been discovered to be involved in the pathogenesis mechanism of OS and may serve as an oncogenic lncRNA. Chan LH et al. demonstrated that lncRNA H19 is overexpressed by the up-regulation of Hedgehog signaling and overexpression of Yap1, and the aberrant Hedgehog signaling contributes to the tumorigenesis of OS [60]. In another study, Peiheng He et al. revealed that overexpression of tumor suppressor miR-141 inhibited osteoblastic cell proliferation through down-regulation of H19 or miR-675 in OS [61], which also illustrates the interaction of endogenous miRNAs with lncRNAs.

HOTAIR (HOX transcript antisense RNA, HOTAIR), a 2337nt lncRNA located at 12q13.13, has a high expression in OS tumor tissues, and notably promotes tumor growth [62]. With better study in many other types of cancers including ovarian cancer [63], hepatocellular carcinoma [64], lung cancer [65] and breast cancer [66], HOTAIR was reported to have a role as a molecular scaffold binding PRC2 through the 5′ domain and LSD1/CoREST/REST complexes through the 3′ domain, so as to participate in tumor development by promoting histone H3K27 trimethylation and inhibiting gene expression [67, 68]. But this action mechanism remains to be studied in OS. Additionally, rs7958904, a HOTAIR variant, was markedly associated with lower risk of OS in a two-stage, case-control study [69].

loc285194, located at osteo3q13.31 and acting as a tumor suppressor, was first reported lncRNA in OS by Pasic, I. et al. in 2010 [70]. However, the underlying mechanism of it in OS hasn't been reported. Later on, by using HCT-116 cells, Qian Liu et al. found that a p53-regulated lncRNA loc285194 inhibits cell growth both *in vitro* and *in vivo*. The underlying mechanism of it in colon tumor is partly due to the negatively regulated miR-211and they further revealed a reciprocal suppression between loc285194 and miR-211 [71]. These results indicate the possible functional mechanisms of loc285194 in OS and in-depth exploration of loc285194 in OS is needed.

## LNCRNAS IN INVASION, MIGRATION AND METASTASIS

Metastasis and relapse are principal pathological problems in the malignant progression of OS, which evidently hampers the effectiveness of OS clinical treatmets and brings unfavorable outcomes to OS patients. Acording to the statistics, 90% of OS patients occurred lung metastasis before the application of chemotherapeutics [72] while only 20% of patients with metastasis and recurrence at diagnosis could be cured even with multi-drug treatment [73]. Therefore, high rate of metastasis is a big challenge that should be solved for successful clinical treatments in OS. To achieve it, better understanding of underlying mechanism of tumor invasion, migration and metastasis is of great benefit. In recent years, lncRNAs have been reported to be involved in tumor invasion and metastasis, and presented a brilliant prospect in many different cancers including OS, which may provide valuable therapeutic targets and potential biomarkers for prognosis [74–76].

The expression of ODRUL (OS doxorubicin-resistance related up-regulated lncRNA, ODRUL) was elevated in tumor tissues of OS patients with lung metastasis [77]. Increased HULC (highly up-regulated in liver cancer, HULC) was found to be associated with clinical stage and distant metastasis for OS patients by Xiaohui Sun et al. [78] On the contrary, it showed no potential functions of HULC in clinicopathological characteristics of OS patients in another study [79]. MALAT1 facilitates metastasis in OS, possibly through activation of PI3K/AKT signaling pathway [54]. High expression of HOTAIR notably promotes OS invasion by increasing the secretion of MMP2 and MMP9 [62]. SNHG12 functions in migration by elevating expression of angiomotin gene in human OS cells [45]. PACER promotes metastasis in OS *via* NFκB-dependent activation of COX-2 gene [47]. LncRNA MFI2 could promote migration of OS cells through upregulating FOXP4 expression [46]. Markedly elevated BCAR4 (Breast Cancer Anti-Estrogen Resistance 4, BCAR4) is associated with lung metastasis by activation of transcription of GLI2-dependent gene [80]. Upregulated ZEB1-AS1 has connection with tumor metastasis in OS by the way of increasing ZEB1 expression [48]. Additionally, lncRNAs BANCR (BRAF-activated noncoding RNA, BANCR) [27], and UCA1 (urothelial carcinoma associated 1, UCA1) [81] are associated with distant metastasis and also identified as independent prognostic biomarkers for poor outcomes in OS.

## LNCRNAS IN CELL APOPTOSIS

Induction of cancer cells apoptosis is the ultimate aim in cancer treatment. However, some malignant cancer cells may enhance their viability, reduce cell apoptosis, and then become drug resistant during diverse cancer drug treatments. These drug-resistant cancer cells then contribute to tumor origination and progression, which makes malignant cancers clinically intractable diseases. Some recent studies focusing on lncRNAs have demonstrated involvement of lncRNAs in OS cell apoptosis, which indicates latent therapeutic targets for effective clinical treatments.

Qiang Zhang et al. showed that suppressing expression of TUG1 significantly promoted OS cell apoptosis. [29, 50] Yin Z et al. found that lncRNA MFI2 could promote cell apoptosis by regulating *FOXP4* expression. [46] Z.Q. Peng et al. discovered that inhibition of BANCR promoted MG-63 cell apoptosis *in vitro* in addition to suppressing cell proliferation and invasion. [27] Peiheng He et al. reported that high expression of miR-141 promoted cell apoptosis by reducing lncRNA H19 or miR-675 in OS. [61] In another study, Yunlu Liu et al. elucidated the effects of MALAT1 on cell apoptosis induction *via* the RhoA/ROCK pathway to facilitate the tumorigenesis of OS. [55]

Collectively, these lncRNAs facilitate development and progression of OS by participating in cell growth, proliferation, invasion, migration, metastasis and cell apoptosis. These study findings present potential clinical values of lncRNAs to be therapeutic targets for effective OS treatments. The concrete action mechanisms in many of them are still unexplored, thus in-depth studies remain to be performed.

## POTENTIAL UTILITY OF LNCRNAS IN OS

### LncRNAs in OS diagnosis and prognosis

In recent decades, the treatment effectiveness for OS patients hasn't been improved even with much more therapeutic choices. OS is apt to metastasis and recurrence, which causes unpleasant clinical outcomes. Early diagnosis and accurate prognosis may make it possible to take timely treatment and clear focus in the early stage, and thus have contribution to enhance the overall survival of OS patients. Therefore, appropriate biomarkers for diagnosis or prognosis have a great clinical significance. Research results about lncRNAs associated in OS indicate that lncRNAs may have potential clinical values as diagnostic or prognostic biomarkers.

Overexpression of lncRNA FGFR3-AS1 (Fibroblast Growth Factor 3 antisense transcript 1, FGFR3-AS1) is correlated with large tumor size, advanced Enneking stage, and poor survival [82]. Increased HOTTIP (HOXA transcript at the distal tip, HOTTIP) was recently discovered in human OS samples, connecting with advanced clinical stage, metastasis and poor prognosis. [83] As previously mentioned, there are contradictory results in clinicopathological characteristics of OS patients in two studies, but both studies demonstrated that overexpression of HULC was correlated with unfavorable clinical outcomes. [78, 79] Distinctly upregulated BCAR4 is associated with large tumor size, advanced clinical stage, and poor outcomes *via* activating transcription of *GLI2*-dependent gene. [80] Overexpressed ZEB1-AS1 has close relations to large tumor size, progressed Enneking stage, and poor overall survival in OS through activation of ZEB1 transcription [48]. Decreased lncRNA MEG3, which positively regulates the expression of *p53* gene [84], was found to be correlated with unfavorable overall survival of OS. [85] Significantly higher expression of lncRNA FOXC2-AS1 was associated with longer survival time for OS patients. [20] These findings indicate the promising ability of lncRNAs to be possible biomarkers for prognosis in OS clinical treatments.

loc285194 was first discovered in OS but the underlying mechanism is totally unclear. However, Qian Liu et al. reported the low expression of loc285194 detected in colon tumor specimens, and indicated that loc285194 could be a diagnostic marker for colon cancer. [71] Therefore, the potential diagnostic role of loc285194 in OS remains to be explored.

Additionally, some explored lncRNAs have been identified as independent prognostic biomarkers in OS. BANCR (BRAF-activated noncoding RNA, BANCR), which is 693bp in length, was initially discovered in melanoma cells by Flockhart et al. in 2012. [86] Elevated BANCR was markedly correlated with large tumor size, advanced clinical stage, distant metastasis and poor survival in OS and it was suggested by Z.Q. Peng et al. to be an independent predictor of poor prognosis. [27] TUG1 (Taurine Up-regulated Gene 1, TUG1), which was reported to be a possible therapeutic target in OS [50], also has close connection with disease status, for which plasma TUG1 may contribute to OS diagnosis, prognosis and dynamic surveillance. [87] Upregulated UCA1 (urothelial carcinoma associated 1, UCA1) could be connected with large tumor size, advanced clinical stage and distant metastasis, and serve as an independent prognostic biomarker for poor outcomes. [81] Increased expression of lncRNA 91H (antisense H19 transcript, 91H) not only was notably associated with large tumor size, advanced clinical stage and post-surgery chemotherapy, but also could be an independent predictive factor of poor survival for OS patients. [88] Higher expression of MALAT1 existed in OS patients with advanced clinical stage and distant metastasis, and contributed to a shorter survival time, which illustrates its potential as an independent prognostic factor to predict patients’ survival situation [89].

### LncRNAs in OS chemotherapy

The application of chemotherapy has provided revolutionary changes to the survival rate of OS patients. [90] However, there are still lots of OS patients having less sensitivity to current chemotherapeutics like doxorubicin and cisplatin, which subsequently results in poor clinical outcomes. [14] In recent three decades, the overall survival of OS patients seems to have stagnated, which urgently requires seeking for novel therapeutic targets and effective reversion of resistance to current therapeutic drugs.

As for novel therapeutic targets, researchers have made a tentative exploration in the involvement of lncRNAs in OS in recent years. These oncogenic or tumor suppressive lncRNAs that participate in OS pathogenesis, provide potential therapeutic targets for OS clinical treatments. For example, overexpressed HOTAIR in OS tumor tissues that markedly promotes tumor growth and metastasis [62], increased MALAT1 with involvement of signaling pathways and miRNA [54–56], down-regulated lncRNA TUSC7 associated with cell proliferation [43], elevated BCAR4 related to lung metastasis by regulating GLI2-dependent gene [80], and independent prognostic predictor BANCR [86] imply the possiblity of targeted therapy based on lncRNAs.

Cancer stem cells (CSCs) with stem cell-like properties, account for a small part in malignant cancers, but they take responsibility for cancer initiation and progression. [4, 91, 92] It is reported that CSCs also exist in OS and contribute to tumor invasion and relapse. [93] Therefore, treatments targeting at CSCs may obtain some unexpected improvements for OS patients. For achieving this, a good understanding of CSCs is absolutely needed. Yongcheng Wang et al. were the first to explore the role of lncRNAs in CSCs. They demonstrated that upregulated HIF2PUT (hypoxia-inducible factor-2α promoter upstream transcript, HIF2PUT) significantly suppressed cell proliferation and migration, reduced the percentage of CD133 expressing cells, and damaged the OS stem sphere-forming ability of the MG63 cells. Besides, HIF2PUT had positive relationship with the expression of *HIF-2α*. This study provides a hint that HIF2PUT may be a useful regulatory target of OS stem cells as well as a latent therapeutic target for OS treatments [94].

As for reversion of resistance to therapeutic drugs, plentiful studies have focused on exploring the underlying mechanisms of chemoresistance in OS by genetic and molecular analyses, and tremendous progress has been made. Findings from these studies present diverse biological alterations, including up-regulation of ABC membrane transporter family members, abnormal metabolic pathways, interference in cell cycle regulation and disorders of cell death pathways. [95–97] As a result, these changes can cause drug inactivation [98], reduced intracellular drug accumulation [99], cancer stem cell (CSC)-mediated chemoresistance [100, 101], disorders in signal transduction pathways [102], dysregulation of miRNAs [103, 104] as well as apoptosis- and autophagy-related drug resistance [105, 106].

However, almost none of the currently available approaches can effectively reverse the poor chemoresponse in OS. Chemoresistance now extremely hampers the efficacy and improvement of clinical therapies for OS patients. Hence, better understanding of the underlying mechanism of chemoresistance from a new prospective may provide feasible methods to effectively reverse drug resistance in OS and improve overall survival of OS patients. In view of the existence of much more lncRNAs compared to miRNAs [21], research focus is shifted to their unclear functions. Therefore, despite of well-studied miRNAs in resistance to chemotherapeutics [103, 104], researchers recently have paid attention to the function of unexplored lncRNAs.

A recent study demonstrated that a distinctly higher expression of lncRNA ENST00000563280, which was named FOXC2-AS1 (FOXC2 antisense RNA 1, FOXC2-AS1), was associated with poor chemoresponse for OS patients, implying its potential to be a novel predictor of chemoresponse. It was also revealed that FOXC2-AS1 may have contribution to doxorubicin resistance by increasing the expression of some classical MDR (multidrug resistance) associated genes, including *ABCB1*and *HIF1A*. [20] Overexpression of ODRUL (OS doxorubicin-resistance related up-regulated lncRNA, ODRUL) was shown in tumor tissues of OS patients with lung metastasis and a low chemoresponse. It was reported that lncRNA ODRUL may reduce sensitivity to doxorubicin in OS cells by inducing expression of *ABCB1*, which is classically related to multidrug resistance. [77] LncRNA LINC00161 (long intergenic non-coding RNA 161, LINC00161) was revealed to play an essential role in cisplatin-induced apoptosis, and attenuate OS chemoresistance by targeting the miR-645-IFIT2 (interferon-induced with tetratricopeptide repeats 2, IFIT2) signaling axis. [107] Upregulated HOTTIP was recently discovered in human OS specimens, and associated with advanced clinical stage, metastasis and poor prognosis. [83] Previously, the action mechanism of HOTTIP was elucidated in Hirschsprung disease, in which HOTTIP interacted with WDR5/MLL complex to increase the expression of multiple 5′ HOXA genes by enhancing histone H3 lysine 4 trimethylation. [108] But this mechanism hasn't been demonstrated in OS. Of note, HOTTIP also participated in OS cellular resistance to cisplatin by the activation of Wnt/β-catenin signaling pathway, which indicates a potential therapeutic approach to targeting Wnt/β-catenin signaling pathway to reverse the resistance. [109]

In summary, lncRNAs possess potential of serving as diagnostic or prognostic biomarkers, and providing viable therapeutic targets. Drug resistance associated lncRNAs directly connect drug resistance with intracellular MDR associated genes, signaling pathways and interaction with miRNA, which further complements and optimizes our present understanding of OS chemoresistance mechanisms. The clinical application of these lncRNAs still requires further identification and verification.

## SUMMARY AND PROSPECT

This review summarizes results from recent studies of lncRNAs that act as oncogenes by overexpression or tumor suppressors by downexpression in OS, like HOTAIR, MALAT1, H19, TUG1, MEG3 and TUSC7. By serving as pivotal regulators, they participate in the pathogenesis process of OS, including cell growth, proliferation, invasion, migration, metastasis and cell prognosis. Several mechanisms are found from these lncRNAs, such as targeting some associated host genes, involvement of signaling pathways, competing endogenous RNA and so on. In addition, some lncRNAs are identified as independent prognostic biomarkers and some are involved in resistance to currently available chemotherapeutics like doxorubicin and cisplatin. These results show great promise for developing feasible diagnostic or prognostic biomarkers and prospective therapeutic targets based on lncRNAs. Further investigation and identification are still needed for these lncRNAs. Besides, in order to apply them to clinical diagnosis and treatment, it's inevitable to study the effects of them in human beings by carrying out large-scale clinical trials in the near future.

Despite of the above achievements, here we would also like to put forward some problems and puzzles based on these recent studies.

First, where can we get samples to detect lncRNAs for human OS patients? Clinically, it's difficult and inconvenient to obtain tumor tissues for early diagnosis and prognosis. Researchers commonly get tissue samples after surgery resections. Of note, a recent study detected plasma lncRNA TUG1and showed a strong correlation between the level of plasma lncRNA TUG1 and disease status. [87] We predict that liquid specimens, such as human peripheral blood, may commendably solve this problem and provide real-time dynamic surveillance. Besides, for patients with lung metastasis, sputum possibly becomes a better choice.

Second, overexpression of BANCR is detected in OS patients [27], but it's still positively associated with low viability of OS cells in a baicalein-treated process. [110] Baicalein, a bioactive flavonoid pervasively used in ancient China, was newly reported to have an effect on apoptosis, cell cycle arrest, migration and invasion of OS in 2013. [111] These contradictory findings may imply that BANCR can function by different mechanisms in different situations. Or maybe the increased BANCR in a baicalein-treated process aims to resist the effect of baicalein. To make it clear, further investigation of BANCR in OS is requested.

Third, whether lncRNAs can be induced in the process of treating therapeutic agents is needed to be identified. 17β-estradiol (E2) has been put forward to play a role in inhibition of OS cell proliferation since 1970s [112], but it hasn't been applied to OS clinical therapy. Recently, a study revealed that after treating with high dose of E2 in OS cells, the expression of miR-9 was elevated, which subsequently reduced the expression of MALAT1 at posttranscriptional level. [53] As mentioned earlier, MALAT1plays an oncogenic role in OS. [54] The decreased MALAT1 after E2 treatment and increased BANCR after baicalein treatment may lead to a guess that lncRNAs can be induced after treatment of therapeutic agents.

Though the above research findings present a good tendency of research development, the research progress of lncRNAs in OS is still preliminary. Therefore, much more time and efforts should be invested in exploring a more comprehensive mechanism of lncRNAs in OS pathogenesis, chemoresistance as well as therapeutic agents treatment. Researchers are confronted with numerous challenges, such as difficulty of obtaining substantial tumor specimens because of the low morbidity, extensive heterogeneity between or in tumor tissues and a fairly complex genetic background. Not until these challenges are overcome can we have a better understanding of molecular mechanisms in OS and achieve a goal of effectively improving overall survival of OS patients.

We anticipate that lncRNAs will be better studied in OS and play a crucial role in clinical diagnosis or prognosis and treatment of human OS before long. LncRNA-targeting therapies will offer unexpected and overwhelming benefits for OS patients.
